# Relationships Among Functional Status, Global Self-Reported Categorical Measure of Activity Level, Health-Related Quality of Life and Psychological State in Patients with Parkinson’s Disease in Greece

**DOI:** 10.3390/brainsci16010090

**Published:** 2026-01-15

**Authors:** Anna Christakou, Nektaria Angeliki Komisopoulou, Amalia Panagiota Louka, Vasiliki Sakellari

**Affiliations:** 1 Laboratory of Biomechanics, Department of Physiotherapy, University of Peloponnese, 23100 Sparta, Greece; 2 Department of Physiotherapy, School of Health Sciences, University of West Attica, 12243 Athens, Greece; nektaria.komissopoulou@gmail.com (N.A.K.); amlouk9@gmail.com (A.P.L.); vsakellari@uniwa.gr (V.S.)

**Keywords:** Parkinson’s disease, activity level, functional status, health-related quality of life, psychological state

## Abstract

**Background/Objectives:** Parkinson’s disease is the second most common neurodegenerative disorder, affecting patients’ daily lives in multiple domains, including functional status, health-related quality of life, and psychological well-being. This study examined the relationship between self-reported global activity level, functional status, Health Related QoL (HRQoL), and psychological state among patients with Parkinson’s disease in Greece. **Methods:** Thirty volunteers (mean age = 69.07, SD = 11.24), members of the Greek Parkinson’s Patients and Caregivers Association, completed (a) the Parkinson’s Disease Questionnaire to evaluate HRQoL and (b) the Hospital Anxiety and Depression Scale (HADS) to assess psychological state. Participants then performed (a) the Five Times Sit to Stand Test (FTSST) and (b) the Berg Balance Scale (BBS) to evaluate functional status. All questionnaires and the test used in the present study have been validated in Greek. Correlation analysis with Spearman r tests with Bonferroni correction was performed between the above variables. Subsequent linear regression models were used to identify independent predictors of HRQoL and balance using SPSS 29.0.2.0. **Results**: Participants reported elevated anxiety (M = 9.67, SD = 4.44) and depressive symptoms (M = 8.97, SD = 4.08), alongside relatively high HRQoL scores (M = 40.09, SD = 18.40). Impaired functional performance was observed, with 22 participants failing to complete the FTSST within 16 s and 16 scoring below 40 on the BBS. Functional status was strongly correlated with HRQoL (r = −0.696, *p* < 0.001) and activity level (r = −0.521, *p* < 0.008). Depression was also significantly associated with poorer HRQoL (r = 0.618, *p* < 0.008) and lower activity levels (r = −0.545, *p* < 0.008). Regression analyses revealed that balance (β = −0.526), disease duration (β = 0.437), anxiety (β = 0.411), and lower limb function (β = −0.351) were significant independent predictors of HRQoL (R^2^ = 0.785; F(9, 20) = 12.69; *p* < 0.001), while HRQoL (β = −0.738) and lower limb function (β = −0.391) independently predicted balance (R^2^ = 0.699; F(9, 20) = 4.72; *p* = 0.002), suggesting a bidirectional relationship between physical function and subjective well-being. **Conclusions:** Activity level, HRQoL, functional status, and psychological state in patients with Parkinson’s disease are interrelated factors. Increased levels of anxiety and depression, as well as reduced HRQoL, were observed. The findings point to a potentially reinforcing cycle between poor balance and diminished quality of life, with anxiety and age playing key roles. Overall, the results illustrate that functional, psychological, and HRQoL measures interact in complex ways, emphasizing the multidimensional profile of patients with Parkinson’s disease. Further studies with larger samples are required to confirm these findings.

## 1. Introduction

Parkinson’s disease (PD) is one of the most common movement disorders and is the second most prevalent neurodegenerative disease of the central nervous system [1,2]. The onset of the disease is attributed to the loss of dopaminergic neurons or to the accumulation of alpha-synuclein (αSyn), which is found in Lewy bodies [3]. The disease is characterized by the presence of motor symptoms, such as bradykinesia, resting tremor, rigidity, and balance impairment [4], as well as non-motor symptoms [1]. Together, these symptoms substantially impair mobility and the ability to perform activities of daily living, impose a significant psychological burden, and lead to a marked deterioration in health-related quality of life (HRQoL) [2].

According to the Global Burden of Disease [5], PD affects an estimated 6.2 million individuals worldwide, and based on research data, the number of patients is expected to double by 2040, reaching 12 million. In Europe, the number of patients with PD is approximately 1.2 million and is projected to reach 3 million by 2030. Evidence indicates that the prevalence of the disease increases over time, and it is expected to affect 2% of individuals over 60 years of age and 6% of individuals over 80 [6]. The cost per patient with PD averages approximately €11,000 across Europe, while the total annual cost for European countries amounts to €13.9 billion [7]. Additionally, the cost per patient rises as the disease progresses, since non-motor symptoms constitute a significant cause of hospitalization [8].

PD is characterized by progressive deterioration, which results in a steady decline in patients’ HRQoL due to reduced functional status. Kilinc et al. [9] examined the quality of life in relation to functional status and tremor in patients with PD, and they found that quality of life is associated with upper-limb functional status and tremor severity. A review investigating HRQoL in patients with PD showed that reduced mobility and inability to perform daily activities are directly associated with HRQoL [10].

HRQoL in PD reflects a complex and multifactorial construct shaped by the interaction of motor, psychological, and behavioral factors. Physical activity (PA) has emerged as a particularly important and potentially modifiable determinant, with higher activity levels associated with improvements in motor performance, psychological well-being, functional status, and HRQoL [11]. Patients with PD consistently report lower HRQoL than healthy individuals across most domains, especially physical functioning and psychological well-being, highlighting the combined burden of motor and non-motor impairments [12]. Notably, psychosocial factors—most prominently depression—appear to exert an even greater influence on HRQoL than motor symptoms in many studies, with anxiety, apathy, and pain often demonstrating stronger associations with reduced HRQoL than motor severity alone [12]. Depression, the most common non-motor symptom, affects approximately 31.8% of patients, while anxiety is present in about 21.8%, with prevalence influenced by disease stage and motor symptom severity [13]. Although most studies report no sex-related differences in the occurrence of depression, some findings suggest a higher prevalence among women [14].

Reduced PA is a well-recognized feature of PD, arising from both motor impairments and non-motor symptoms. Compared with healthy controls, patients with PD spend significantly less time engaged in daily activities and exhibit an estimated 29% reduction in overall movement and energy expenditure [15]. Lower activity levels are associated with greater motor severity, increased disability in activities of daily living, and poorer functional performance [16]. Conversely, higher levels of PA are linked to more favorable motor and non-motor outcomes, including lower depressive scores and improved HRQoL, underscoring PA as both a marker of disease burden and a key therapeutic target for enhancing overall well-being in this population [17].

Despite the growing body of international literature, comprehensive investigations examining how self-reported activity level, functional status, and psychological state jointly relate to HRQoL in Greek patients with PD remain limited. Previous research in Greece has focused primarily on the validation of the PDQ-39 [18] and on isolated determinants of HRQoL, such as comorbidities, functional dependence, and depression [19]. However, these studies have not examined the integrated relationships among global activity level, functional status, psychological state, and HRQoL within the Greek PD population. Given that cultural, lifestyle, and healthcare system factors may influence activity patterns, coping strategies, access to rehabilitation services, and psychosocial support, findings from other populations may not be directly generalizable to Greek patients. A context-specific understanding of these interrelated determinants is therefore essential for informing targeted interventions, optimizing individualized care pathways, and supporting evidence-based physiotherapy and psychosocial management within the Greek healthcare setting.

It is also important to note that for instruments such as the PDQ-39, higher scores indicate worse HRQoL; this scoring directionality directly informs the study hypotheses, whereby greater depressive symptomatology, lower activity levels, and impaired functional status are expected to be associated with higher (worse) PDQ-39 scores [18].

Accordingly, the present cross-sectional study aimed to examine the relationships among self-reported global activity level, functional status, psychological state, and HRQoL in Greek patients with PD. By integrating functional and psychological assessments within a culturally specific sample, this study seeks to clarify how these interrelated determinants contribute to HRQoL, with implications for physiotherapy, psychosocial support, and comprehensive PD management strategies tailored to the Greek population.

The hypotheses of the present study were as follows:

**H1.**  *Functional status is negatively correlated with PDQ-39 (r < 0)*.

**H2.**  *Functional status is positively correlated with activity level (r > 0)*.

**H3.**  *Activity level is negatively correlated with HADS (r < 0)*.

**H4.**  *Functional status is negatively correlated with psychological distress as measured by HADS (r < 0)*.

**H5.**  *PDQ-39 scores are positively correlated with psychological distress (r > 0)*.

The present study is organized as follows: Section 2 describes the materials and methods, including the study design, participant recruitment, and eligibility criteria, assessment instruments, procedures, and statistical analyses. Section 3 presents the results of the study, including descriptive characteristics of the sample and the associations among functional status, psychological variables, activity level, and HRQoL. Section 4 discusses the findings in relation to existing literature, addresses clinical implications, limitations, and future research directions, and Section 5 summarizes the main findings of the study.

## 2. Materials and Methods

### 2.1. Design

This study had a cross-sectional research design and was conducted and reported in accordance with the STROBE (Strengthening the Reporting of Observational Studies in Epidemiology) guidelines [20]. The study was also in agreement with the Declaration of Helsinki ethics principles.

### 2.2. Sample

Using G*Power 3.1 (Exact test family, correlation: bivariate normal model), with α = 0.05 (two-tailed) and a desired statistical power of 1 − β = 0.80, the a priori analysis indicated that a minimum sample of N = 29 participants would be required to detect the hypothesized correlation. To account for rounding and possible missing data, the sample size was set at 30 participants.

Thus, 30 participants were recruited in Athens, Greece, through the Parkinson’s Patients and Caregivers Association from June 2024 to August 2024. Of the 90 individuals informed about the study, 35 expressed interest, and 30 met eligibility criteria and completed all assessments. The reasons for non-participation were not systematically recorded. Participants were recruited exclusively through the Greek Parkinson’s Patients and Caregivers Association, which may introduce systematic selection bias. Members of patient associations might differ from the general PD population in terms of disease awareness, health-seeking behavior, or engagement in rehabilitation activities. This potential bias is considered in the interpretation of findings and generalizability. The neurologist associated with the Parkinson’s Patients and Caregivers Association permitted participation only for individuals with mild-to-moderate PD. This restriction was implemented to ensure participant safety during functional assessments and to maintain a relatively homogeneous clinical profile with respect to disease severity. Disease severity stratification was not performed on the sample. The assessments were conducted under standardized conditions and at a consistent time of day. The time of assessment was set at 17:00, as participants had taken their prescribed PD medications 1–2 h beforehand, a timeframe that has been shown to be optimal for exercise in this population [21]. Patients were instructed to wear comfortable clothing and athletic shoes. The assessments were conducted by two experienced physiotherapists to ensure standardized administration and reliable measurement of functional outcomes.

Participants were eligible for inclusion if they

(a) Were over 50 years of age;
(b) Had a confirmed diagnosis of PD;
(c) Presented with bilateral motor involvement without postural instability or with mild postural instability while remaining physically independent;
(d) Identified Greek as their primary language of communication;
(e) Were ambulatory.

Individuals were excluded if

(a) They exhibited very mild unilateral symptoms without functional impact;
(b) They exhibited advanced disease characterized by severe postural instability; marked gait impairment, or dependence on physical assistance for standing or walking;
(c) They demonstrated an inability to follow simple instructions to the extent that the presence of a companion was required;
(d) Personal or others’ safety could be compromised;
(e) They presented with an unstable health condition.

### 2.3. Instruments

The following valid assessment tools were used:

Parkinson’s Disease Questionnaire (PDQ-39) [18,22]

The PDQ-39 is a self-administered questionnaire that reflects the full spectrum of HRQoLin individuals with PD [23]. It has been translated for Greek patients with PD [18] and is used more frequently than other questionnaires for patient-reported HRQoL assessment in PD [24]. The questionnaire demonstrates high internal consistency and validity, with Cronbach’s α = 0.84 [22]. It consists of 39 items divided into eight dimensions: mobility (10 items), activities of daily living (6 items), emotional well-being (6 items), stigma (4 items), social support (3 items), cognition (4 items), communication (3 items), and bodily discomfort (3 items). Each question offers five response options: never, occasionally, sometimes, often, and always. The scores from all items are summed to yield a total score, which represents the overall HRQoL.

Hospital Anxiety and Depression Scale (HADS) [25,26]

The Hospital Anxiety and Depression Scale (HADS) is a fourteen-item instrument used to assess anxiety and depressive symptoms in patients. Its purpose is to provide clinicians with an acceptable, reliable, valid, and easy-to-use practical tool for identifying and quantifying depression and anxiety [26]. It consists of two subscales of seven items each—one for anxiety (HADS-A) and one for depression (HADS-D). Patients rate each item on a scale from 0 (“Not at all”) to 3 (“Occurs frequently”), with total scores ranging from 0 to 21. HADS demonstrates high accuracy in detecting depression and anxiety in patients with movement disorders. The HADS has been translated into Greek and applied to hospital patients [23]. The scale shows high internal consistency (α = 0.884), specifically α = 0.829 for anxiety and α = 0.840 for depression, as well as a high reliability index ICC = 0.944 [27].

Five-Time Sit-to-Stand Test (FTSST) [28]

This test is used to assess lower-limb functional capacity in older adults and also identifies fall risk and functional ability. To perform the test, the individual must sit with their back touching the chair, feet shoulder-width apart, and arms crossed at chest level. The participant must stand and return to the seated position without touching the backrest five times as quickly as possible. The FTSST has well-documented reliability and validity in older adults and various clinical populations, including individuals with PD, with studies reporting high intra-rater and test–retest reliability (ICC values approximately 0.89–0.96 to 0.95) and satisfactory correlations with other established measures of functional mobility [29] with Cronbach’s α equal to 0.99 [30]. In patients with Parkinson’s, performance is affected by core disease symptoms, such as balance impairment and bradykinesia. The test is recommended for patients with mild-to-moderate symptoms [31].

Berg Balance Scale (BBS) [32]

The Berg Balance Scale (BBS) is used in 30% of studies, often in combination with other assessment tools. The BBS evaluates dynamic and static balance and functional ability to detect high fall risk and predict complications such as fractures that may occur after falls. The tasks include activities such as sitting and standing unsupported, transferring between seated and standing positions, reaching forward while standing, retrieving an object from the floor, turning to look over each shoulder, rotating 360 degrees, placing each foot alternately on a step, and standing in tandem and single-leg stances. Performance on each item was scored on a 5-point ordinal scale ranging from 0 (inability to complete the task) to 4 (independent and optimal execution), with higher scores indicating better balance ability. It was initially developed for older adults but was later applied to other populations, including amputees and patients with PD. It consists of fourteen tasks, scored on a five-point scale from zero to four, which are then summed. A score below forty-five indicates a high fall risk [33]. The scale has an established threshold of a score below 40, indicating high fall risk in individuals with Parkinson’s disease [33]. The scale demonstrates high reliability and validity, with Cronbach’s α > 0.90 [34]. The BBS was translated into Greek and validated by Lampropoulou et al. [35].

Assessment of the Global Self-Reported Categorical Measure of Activity Level

Physical activity (PA) is defined as any bodily movement produced by skeletal muscles that results in energy expenditure and includes activities undertaken during daily living, transportation, occupational tasks, and leisure time [36,37]. In individuals with PD, PA levels are typically reduced due to the combined effects of motor symptoms (e.g., bradykinesia, rigidity, postural instability) and non-motor features such as fatigue, depression, and apathy. PA can be assessed using objective methods, such as accelerometers and wearable sensors, or subjective approaches, including questionnaires and self-report measures. Although objective monitoring provides precise information regarding movement quantity and intensity, it may be limited by cost, participant burden, compliance issues, and feasibility constraints, particularly in older adults and clinical populations. Consequently, brief self-report measures are frequently used in epidemiological and clinical studies to capture habitual activity levels in PD [38]. In the present study, PA was assessed using a single-item self-report categorical question asking participants to rate their overall activity level as low, moderate, or high. The use of a brief global self-reported categorical measure of activity level has precedent in PD. Single-item measures or very short activity questions have been shown to provide meaningful information and are commonly applied when objective monitoring or extensive questionnaires are impractical [36]. Moreover, previous research has demonstrated that short self-report PA measures can offer acceptable validity and utility for epidemiological and clinical research, especially when the goal is to categorize overall activity level rather than quantify precise energy expenditure [37]. This categorical single-item measure provides a global estimate of habitual PA and allows for a coarse categorization of activity level rather than detailed quantification. Accordingly, it does not capture activity intensity or duration and may be subject to recall bias and subjective interpretation. These limitations were considered in the interpretation of the study findings.

### 2.4. Procedure

Initial contact with prospective participants was established through online communication to provide information regarding the purpose and significance of the study. Participant selection required a confirmed diagnosis of PD by a neurologist. Voluntary agreement to participate was a prerequisite for inclusion in the study. The initial meeting was conducted online, was brief in duration, and included a presentation of the study objectives, along with an explanation of the assessments and questionnaires to be administered. All collected data remained anonymous.

During the second meeting, conducted in person, participants first completed the consent form and a demographic information sheet. To minimize measurement bias, all functional assessments were administered by experienced physiotherapists under standardized conditions. Self-report questionnaires were completed with neutral supervision to reduce interviewer influence. Nevertheless, recall and self-report bias cannot be excluded. The patients were asked to rate their activity level in their everyday lives. PA was assessed using a brief single-item self-report measure. Participants were asked, “How would you describe your activity level?” and the participants selected one of three response options: low, average, or high. We chose this concise global rating approach to minimize respondent burden while capturing an overall estimate of habitual activity, which is particularly relevant in PD populations where fatigue, motor symptoms, and cognitive load may limit the feasibility of longer or more detailed questionnaires.

Each participant was then given instructions on the FTSTS. For the assessment to be conducted, the participant was seated on an armless chair with their back resting against the backrest, feet positioned shoulder-width apart, and arms crossed at chest level. The participant was instructed to stand up and return to the seated position five times as quickly as possible, without allowing their back to make contact with the backrest during the repetitions. This part of the assessment lasted approximately 30 s to 2 min. After its completion, participants were informed about the BBS. For the administration of the BBS, each participant completed a standardized series of 14 functional balance tasks designed to evaluate both static and dynamic balance abilities. Each task was demonstrated by the evaluator and then performed by the participant under supervision to ensure safety. The completion of the BBS lasted approximately 15 to 20 min.

Additionally, two questionnaires, the PDQ-39 and the HADS, were administered. For the administration of the PDQ-39, each participant completed the standardized self-report instrument designed to assess HRQoL in individuals with PD. Participants were instructed to reflect on their experiences over the previous month and to rate the frequency of specific difficulties on a 5-point Likert scale. The evaluator provided clear instructions and remained available to address any questions to ensure accurate and complete responses, while maintaining a neutral stance to avoid influencing participant answers. Completion of the questionnaire typically required approximately ten minutes. For the administration of the HADS, participants were instructed to evaluate how they had been feeling over the past week and to select the response option that best reflected their experience on a 4-point Likert scale specific to each item. Clear instructions were provided prior to completion, and the evaluator remained available to address procedural questions without influencing participant responses. The questionnaire typically required approximately ten minutes to complete. The total duration of the assessment session for each participant was approximately 40 min.

### 2.5. Statistical Analysis

Normality of the study variables was examined to determine whether parametric or non-parametric analyses would be conducted, using the Kolmogorov–Smirnov test and the Shapiro–Wilk index. For continuous variables, descriptive statistical analyses were performed, calculating means (M) and standard deviations (SDs).

For correlations between quantitative variables, and specifically for associations between functional status, HRQoL, and psychological state in patients with PD, Spearman’s correlation coefficient (r) was used, as the variables were determined to be non-normally distributed. Data were recorded and analyzed using the Statistical Package for the Social Sciences^®^ (SPSS), version 29.0.2.0. The threshold for statistical significance was set at α = 0.05. We also applied Bonferroni correction to multiple Spearman correlation analyses between activity level, PDQ-39, HADS-A and HADS-D, FTSTS, and BBS, thus changing alpha to 0.05/6, where *p* = 0.0083, in order to control the probability of making at least one type I error across the multiple correlations. Effect sizes were interpreted using Spearman’s r, following Cohen’s guidelines (small ≥ 0.10, medium ≥ 0.30, large ≥ 0.50) [39].

Finally, linear regression was employed to investigate the multivariate relationships between the study variables, specifically modeling HRQoL and balance as outcomes predicted by functional, psychological, activity-related factors, and age, gender, years of PD diagnosis, and number of falls.

### 2.6. Ethics Approval

We obtained approval from the Ethics Committee of the University of West Attica; an application was submitted under protocol number 49016/18th June 2024. Participant anonymity was ensured through pseudonymization of personal data, as well as all collected information used for statistical analyses.

## 3. Results

Table 1 displays the descriptive statistics of the sample. A total of thirty individuals (17 females, 13 males, mean age = 69.07, SD = 11.23) were included. The mean time since diagnosis was 7.23 years (SD = 5.3). There were no missing data for any variables included in the analysis.

Eighteen participants did not have a caregiver, twenty-three were unemployed, twenty-four were not smokers, and twenty of them had experienced a fall since last year. Furthermore, the results indicate that twelve of the participants had a low activity level, fourteen were at a moderate level, and four of them demonstrated a high level of physical activity (Table 2).

**Figure 1:**
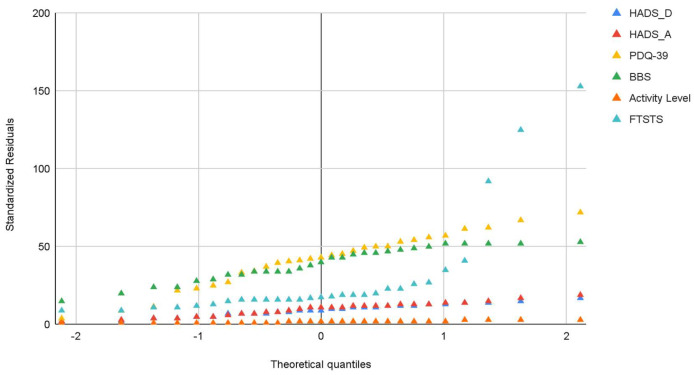
Normal Q–Q plots for the variables.

To assess the distributional properties of the study variables, normality was evaluated using the Kolmogorov–Smirnov and Shapiro–Wilk tests. Both tests indicated statistically significant deviations from normality for the balance and self-reported global activity level variables, whereas the variables related to health-related quality of life (HRQoL), depression, anxiety, and functional status did not show significant departures from normality. Given the presence of non-normally distributed variables and the ordinal nature of the self-reported activity level measure, non-parametric statistical methods were deemed appropriate. Therefore, Spearman’s rho correlation coefficient was used to examine the associations between the total scores of the questionnaires and performance-based tests. Normality testing results for all study variables are presented in Table 3. In addition to formal statistical tests, distributional assumptions were further examined through visual inspection of Q–Q plots. Figure 1 illustrates the Q–Q plots of the six study variables plotted against the theoretical normal distribution.

Table 4 presents the descriptive statistics of the HADS, the PDQ-39, the FTSST, and the BBS.

Results showed that twenty participants (M = 9.67; SD = 4.44) presented symptoms of anxiety (66.7%), and nineteen (M = 8.97; SD = 4.08) had symptoms of depression (63.3%). Furthermore, seventeen participants (M = 40.09; SD = 18.4) demonstrated high HRQoL after completing PDQ-39. Twenty-two participants (M = 28.58, SD = 33.87) were unable to complete the FTSTS within 16 s, a cut-off previously suggested for identifying impaired lower-limb function and increased fall risk in PD populations [40]. Sixteen participants scored below 40 on the BBS (BBS; M = 38.07, SD = 11.79), consistent with established thresholds indicating high fall risk in individuals with PD [33].

Spearman’s correlation analysis identified significant associations among study variables (Table 5). PDQ-39 scores were correlated with HADS-A (r = 0.457, *p* = 0.011), HADS-D (r = 0.618, *p* < 0.001), FTSST (r = 0.381, *p* = 0.038), BBS (r = −0.696, *p* < 0.001), and self-reported global activity level (r = −0.407, *p* = 0.026). HADS-D scores were correlated with FTSST (r = 0.441, *p* = 0.015), BBS (r = −0.454, *p* = 0.012), and self-reported global activity level (r = −0.545, *p* = 0.002). FTSST performance was correlated with BBS (r = −0.596, *p* < 0.001) and self-reported global activity level (r = −0.521, *p* = 0.003). After Bonferroni correction (adjusted α = 0.0083), correlations involving PDQ-39–HADS-D, PDQ-39–BBS, HADS-D–activity level, FTSST–BBS, and FTSST–activity level remained statistically significant.

Linear regression analysis was conducted to identify independent predictors of health-related quality of life, as measured by the PDQ-39. The model, which included gender, age, years since PD diagnosis, self-reported global activity level, number of falls, anxiety (HADS_A), depression (HADS_D), balance (BBS), and lower limb function (FTSTS), was highly significant and explained a substantial 78.5% of the variance in HRQoL scores (R^2^ = 0.785). The overall model was statistically significant (F(9, 20) = 12.69, *p* < 0.001). When examining the unique contribution of each predictor while controlling for the others, four factors emerged as significant. Worse balance was strongly associated with lower HRQoL (β = −0.526, *p* = 0.002). Longer disease duration significantly predicted worse HRQoL (β = 0.437, *p* = 0.013). Higher levels of anxiety (HADS_A) were also a significant predictor of worse HRQoL (β = 0.411, *p* = 0.006). Finally, poorer lower limb function (a higher score on the FTSTS) was associated with worse HRQoL (β = −0.351, *p* = 0.030). In contrast, gender, age, self-reported global activity level, number of falls, and depression did not show statistically significant independent relationships with HRQoL in this model.

A subsequent regression analysis was performed to identify factors predicting balance. This model was also significant, explaining 69.9% of the variance in BBS scores (R^2^ = 0.699, F(9, 20) = 4.72, *p* = 0.002). The analysis identified HRQoL (PDQ-39) as the strongest predictor, with worse HRQoL being significantly associated with poorer balance (β = −0.738, *p* = 0.002). Poorer lower limb function (FTSTS) was also a significant independent predictor of worse balance (β = −0.391, *p* = 0.042). No significant independent effects were observed for gender, age, years since diagnosis, self-reported activity level, number of falls, anxiety, or depression.

## 4. Discussion

The present study examined the associations between self-reported global activity level, functional status, psychological state, and HRQoL in Greek patients with PD. It is important to note from the outset that the cross-sectional and correlational design, together with the use of a self-reported activity measure, limits causal inference and direct comparison with objective activity data. Within this context, the study contributes to the literature on the multidimensional impact of the disease. Recent systematic reviews indicate that HRQoL in PD is strongly influenced by motor impairments, with studies utilizing the PDQ-39 reporting findings consistent with those observed in the present study [12,23]. We identify specific contributions of functional and psychological factors in combination with other demographic characteristics in PD.

Regarding our five specific hypotheses, the results supported four of them. In particular, functional status is negatively correlated with HRQoL (H1). This hypothesis was supported, as a significant association was found between functional status and HRQoL, consistent with previous evidence that functional ability in patients with PD influences HRQoL beyond physical limitations, affecting social participation and daily communication [10,11,12,22]. Balance emerged as a significant and strong negative predictor of HRQoL, alongside disease duration, anxiety, and lower limb function, even when controlling for psychological and demographic factors. This suggests that balance dysfunction contributes substantially to explaining poor quality of life in this clinical population. Similar trends have been reported in earlier studies. Duvdevani et al. [11] assessed HRQoL using the PDQ-39, while functional status was evaluated using the 10-Meter Walk Test in combination with the Frenchay Activities Index. Although different functional assessment tools were employed and the study included a larger sample (N = 88), the observed associations followed comparable patterns, indicating a consistent relationship between functional performance and HRQoL rather than identical outcomes across studies. A particularly notable finding was the evidence of a bidirectional relationship between balance and HRQoL. While balance strongly predicted HRQoL, HRQoL also independently predicted balance. This suggests a potential reinforcing cycle in which declines in one domain may exacerbate declines in the other, highlighting the interconnection between physical function and subjective well-being in PD.

Functional status is positively correlated with self-reported global activity level (H2). This hypothesis was supported, as the observed relationship between PA, typically measured by device-based methods (accelerometers), and functional status in this study aligns with findings that lower PA is associated with poorer functional capacity in PD. Reduced engagement in physical activities is correlated with declines in activities of daily living and may exacerbate motor symptoms, such as impaired mobility and balance, further limiting functional independence [41,42]. Objectively measured PA parameters have been linked to key indicators of functional status and HRQoL in PD, independent of disease severity, suggesting that physical inactivity itself may be associated with diminished functional outcomes. Increased sedentary behavior has been related to greater mobility limitations and lower performance on standardized functional tests, emphasizing the bidirectional relationship between PA engagement and functional capacity [11]. The associations observed between lower activity levels and impaired functional performance are consistent with prior literature, including studies using accelerometers. The self-reported global activity level was not a significant independent predictor when controlling for other factors such as HRQoL and age, suggesting that the link between activity and balance in our sample may be indirect or captured by other interrelated constructs. Our study relied on a global, self-reported activity measure, which does not provide precise data on movement quantity or intensity. Therefore, while the correlation supports H2, comparisons with accelerometer-based studies should be interpreted cautiously, acknowledging this methodological difference.

The self-reported global activity level is negatively correlated with HADS (H3). This hypothesis was supported. The relationship between lower activity levels and higher depressive symptoms in our cohort reflects well-established associations between physical inactivity and psychological distress in PD. A systematic review on PA and depression in PD reported that individuals engaging in lower amounts of PA exhibit more severe depressive symptoms compared with more active counterparts, even after accounting for disease severity and other clinical variables [17]. In addition to direct psychological benefits, PA may indirectly buffer against depression by improving self-efficacy, social engagement, and overall HRQoL, further emphasizing the clinical importance of addressing inactivity in PD.

Functional status was not correlated with HADS (H4). This hypothesis was not supported, as functional status assessed via FTSST and BBS did not demonstrate a statistically significant independent correlation with anxiety or depressive symptoms after controlling for other factors. While previous studies have reported links between reduced functional capacity and higher psychological burden in PD [41,43], the present findings suggest that, in this Greek cohort, non-motor symptoms such as anxiety and depression may be influenced more strongly by other determinants—potentially including perceived HRQoL, social support, or individual coping strategies—rather than by objective measures of functional performance alone [42,43]. Methodological factors may also contribute. The relatively small sample size (N = 30) limits statistical power, increasing the likelihood of Type II error and reducing sensitivity to detect subtle associations between functional status and HADS scores. Additionally, functional status was measured using performance-based clinical tests, whereas psychological state was self-reported, introducing a potential discordance between objective and subjective assessments. Therefore, the lack of support for H4 does not contradict the broader literature but highlights the multifactorial nature of psychological symptoms in PD and the need for larger, longitudinal studies incorporating both objective functional metrics and comprehensive psychological assessments.

HRQoL is positively correlated with anxiety (H5). This hypothesis was supported, as the study identified a significant association between depressive symptoms and HRQoL in PD, reinforcing the view that psychological burden is a central component of disease morbidity. Anxiety, alongside balance, disease duration, and lower limb function, emerged as significant independent predictors of HRQoL, highlighting the particular importance of anxiety symptoms for perceived HRQoL in this cohort. Using the HADS-Depression subscale, approximately 63% of participants scored above the established cut-off (HADS ≥ 8), indicating clinically relevant depressive symptoms. Similarly, Liu et al. [43] demonstrated that non-motor symptoms—including depression—may impact HRQoL even more than motor impairments traditionally associated with PD. Their study reported that 98% of patients had at least one non-motor symptom, with depression and anxiety comprising the third most frequent category (28%), highlighting the pervasive nature of psychological disturbances in this population. However, this prevalence is notably higher than that reported by Sujith et al. [13], who identified depressive symptoms in 31.8% of their PD cohort. This discrepancy may reflect differences in assessment tools and cut-offs, disease severity, sample characteristics, or access to mental health services. Importantly, the HADS screens for the presence and severity of depressive symptoms rather than providing a formal clinical diagnosis. Therefore, the higher prevalence observed should be interpreted as reflecting increased symptom burden rather than confirmed depressive disorder. Nevertheless, the elevated depressive symptoms observed in this sample highlight the possibility that depression in PD may remain underrecognized, particularly among patients with greater functional impairment or social isolation. Consistent with previous research, the significant association between depressive symptoms and lower HRQoL underscores the need for systematic psychological screening and integrated management alongside motor-focused interventions. These results highlight the importance of incorporating routine psychological assessments in PD care to improve the detection of depressive symptoms and facilitate timely interventions, ultimately enhancing overall quality of life.

To our knowledge, this study represents one of the first efforts to integrate functional, psychological, and HRQoL assessments in a sample of Greek patients with PD. While previous regional studies examined HRQoL or depression separately [18,19], our work contextualizes these measures alongside functional status and self-reported activity levels, offering an integrative perspective rather than claiming absolute novelty. The use of the BBS in combination with the FTSST captures a wide spectrum of motor symptoms, including tremor, impaired balance due to poor posture, bradykinesia, and rigidity. This combination allows for a comprehensive assessment of functional status in each participant. The two self-report questionnaires provide an overview of patients’ HRQoL and psychological condition. The selected tests and questionnaires exhibit high reliability and strong internal consistency for this population. The examination of associations between functional performance measures and questionnaire-based outcomes contributes to the limited body of Greek research addressing these relationships in PD. Additionally, analyzing associations between HRQoL dimensions and psychological factors, such as anxiety and depression, alongside functional capacity, provides insight into the multidimensional nature of the disease. The findings highlight the interrelation between psychological status, functional performance, activity level, and HRQoL, underscoring the importance of a comprehensive rehabilitation approach rather than focusing solely on motor symptoms. Functional status was assessed using performance-based measures (BBS and FTSST), while HRQoL and psychological status were evaluated using validated self-report questionnaires (PDQ-39 and HADS). This combined use of objective functional assessments and subjective self-report measures allows for a more comprehensive understanding of factors affecting patients with PD. Furthermore, identifying disease duration as a key independent predictor of HRQoL adds a temporal dimension to understanding well-being in PD.

### 4.1. Clinical Implications

The present findings have several important clinical implications. The strong association identified between the BBS and HRQoL highlights the need for rehabilitation programs emphasizing balance training and fall prevention strategies for patients with PD. Through such interventions, patients may improve independence and reduce fear of falling. The observed relationship between functional status and activity level further highlights the clinical value of promoting PA to help maintain functional capacity, although its non-significant role in the multivariate BBS model suggests that interventions should also directly target balance and HRQoL. Evidence indicates that non-motor symptoms do not significantly affect PA, unlike motor impairments [11]. Clinically, this suggests that promoting PA remains valuable even in the presence of non-motor symptoms. Moreover, the observed associations between HRQoL, anxiety, and depression emphasize the importance of early psychological assessment in patients with PD, as Cong et al. [44] reported that addressing depressive symptoms promptly may contribute to improved HRQoL. Specifically, screening for and managing anxiety symptoms may have a particularly strong impact on HRQoL. Recognizing the prominent and potentially bidirectional role of functional status in HRQoL and psychological state positions physiotherapy as a key pillar for holistic patient care and the prevention of further functional decline. Health practitioners may consider integrating validated screening tools for depression and anxiety into routine assessments, given the observed links among psychological symptoms, functional status, activity level, and HRQoL. Such screening may help identify patients who could benefit from additional psychological evaluation or supportive interventions. Furthermore, these findings underscore the value of interdisciplinary collaboration among neurologists, physiotherapists, and psychologists in addressing the multidimensional needs of individuals with PD, with particular attention to disrupting the potential negative cycle between poor balance and diminished quality of life, promoting a comprehensive and patient-centered approach without implying effects on disease progression. Given the cross-sectional design and limited sample size, these clinical implications should be interpreted as hypothesis-generating suggestions rather than definitive guidance for clinical practice.

### 4.2. Limitations

Several limitations should be considered. The convenience sample (N = 30) was recruited via a patient association and may not represent the broader Greek PD population. The gender imbalance (56.7% female) may have influenced psychological outcomes. Disease severity was not formally stratified; participants were limited to mild-to-moderate PD, reducing generalizability to early or advanced stages. Psychological measures (HADS) and HRQoL (PDQ-39) relied on self-report, potentially introducing bias, and activity level was assessed with a single-item measure rather than objective monitoring. Functional capacity was measured with performance-based tests, but no advanced wearable or sensor-based assessments were included. Finally, the cross-sectional design precludes causal inference; the bidirectional relationship between balance and HRQoL warrants longitudinal investigation.

### 4.3. Future Considerations

The complex relationships among functional status, psychological state, and HRQoL in patients with PD remain insufficiently explored, particularly within the Greek population, where living conditions and healthcare access may differ from those in other countries. Future research should aim to include larger, more geographically diverse samples to enhance the generalizability of findings and to examine the influence of demographic factors on these outcomes. Larger samples are necessary to validate the present findings. Future studies should incorporate objective activity monitoring (e.g., accelerometers) to overcome the limitations of self-reported measures and to more accurately quantify associations involving physical activity, building on the correlations observed in this study. Additionally, stratified analyses based on activity level, sex, age, or history of falls could provide valuable insights, as such subgroup comparisons may reveal differential patterns in functional status, psychological burden, and HRQoL. Investigating these clinically relevant subgroups may increase the applicability of the findings by informing more targeted and individualized rehabilitation and prevention strategies for individuals with PD.

Longitudinal studies are recommended to track changes in functional status, psychological well-being, and HRQoL over time, providing insight into the progressive nature of PD and its impact on these variables. Such designs are essential to test the hypothesized reinforcing cycle between balance and HRQoL suggested by our cross-sectional models. Based on the observed associations between functional capacity, activity level, and psychological status in the present study, future investigations could prioritize interventions targeting balance, mobility, and PA as strategies to enhance both functional and psychosocial outcomes.

Additionally, studies should assess the long-term effects of holistic physiotherapy and multidisciplinary rehabilitation programs on functional status, psychological health, and HRQoL. Research is also needed to identify strategies to prevent decline in key functional and psychological domains, integrating both medical and physiotherapeutic approaches. Overall, the present findings provide a foundation for designing targeted interventions and for guiding future research aimed at improving comprehensive care for patients with PD.

## 5. Conclusions

This study investigated the relationships among functional status, psychological state, self-reported global activity level, and health-related quality of life in Greek patients with PD. Significant associations were observed between functional status and HRQoL, as well as between psychological variables and HRQoL. Additionally, self-reported global activity level was linked to both functional performance and depressive symptoms. These findings highlight the interrelated nature of functional, psychological, and HRQoL outcomes in this population. Future research employing larger, more diverse samples and longitudinal designs is warranted to confirm these associations and to further elucidate their causal and temporal relationships.

## Figures and Tables

**Table 1:** Descriptive statistics of the sample.

Variable	Minimum	Maximum	Mean ± SD
Age (years)	50	92	69.07 ± 11.23
Height (m)	1.50	1.92	1.66 ± 0.12
Weight (kg)	48	114	78.00 ± 16.30
Years since diagnosis	1	23	7.23 ± 5.30
Number of falls in the past year	0	100	12.80 ± 23.31

m: meters, kg: kilogram.

**Table 2:** Percentages of the demographic and clinical characteristics of the participants.

Characteristics		N	Percentage (%)
Sex	Female	17	56.7
	Male	13	43.4
Marital Status	Married	18	60
	Single	2	6.7
	Widowed	7	23.3
	Divorced	3	10
Caregiver	Yes	12	40.0
	No	18	60.0
Employed	Yes	7	23.3
	No	23	76.7
Smoking	Yes	6	20.0
	No	24	80.0
Fall in the Past Year	Yes	20	66.7
	No	10	33.3
Self-reported global activity level	Low	12	40.0
	Moderate	14	46.7
	High	4	13.3

**Table 3:** Normality tests for the study variables.

Variable	Instrument	Kolmogorov–Smirnov Statistic	*p* Value	Shapiro–Wilk Statistic	*p* Value
Anxiety	HADS-A	0.151	0.078	0.966	0.435
Depression	HADS-D	0.090	0.200	0.978	0.784
Health-related quality of life	PDQ-39	0.123	0.200	0.963	0.372
Functional status	FTSTS	0.129	0.200	0.934	0.064
Balance	BBS	0.352	<0.001 **	0.543	<0.001 **
Self-reported global activity level	—	0.256	0.040 *	0.787	<0.001 **

HADS-A = Hospital Anxiety and Depression Scale—Anxiety subscale; HADS-D = Hospital Anxiety and Depression Scale—Depression subscale; PDQ-39 = Parkinson’s Disease Questionnaire-39; FTSTS = Five Times Sit-to-Stand Test; BBS = Berg Balance Scale. *p* < 0.05 *, *p* < 0.01 ** indicate statistically significant deviations from normality.

**Table 4:** Descriptive statistics of the study variables.

Variable	Instrument	Minimum	Maximum	Mean ± SD
Anxiety	HADS-A	2	19	9.67 ± 4.44
Depression	HADS-D	1	17	8.97 ± 4.08
Health-related quality of life	PDQ-39	0	72	40.09 ± 18.40
Functional status	FTSST	7	153	28.58 ± 33.87
Balance	BBS	10	53	38.07 ± 11.79

HADS-A, Hospital Anxiety and Depression Scale—Anxiety; HADS–D, Hospital Anxiety and Depression Scale—Depression; PDQ-39, Parkinson’s Disease Questionnaire; FTSST, Five-Time Sit-to-Stand Test; BBS, Berg Balance Scale.

**Table 5:** Spearman’s correlations among health-related quality of life HRQoL measure, psychological variables, functional performance, and self-reported global activity level.

Variable	PDQ39	HADS_A	HADS_D	FTSST	BBS	Self-Reported Global Activity Level
PDQ-39	-					
HADS_A	r = 0.457 *p* = 0.011 95% CI: 0.05, 0.76	-				
HADS_D	r = 0.618 ** p < 0.001 95% CI: 0.32, 0.82	r = 0.312 *p* = 0.084 95% CI: −0.04, 0.64	-			
FTSST	r = 0.381 *p* = 0.038 95% CI: 0.01, 0.67	r = 0.378 *p* = 0.040 95% CI: 0.03, 0.68	r = 0.441 *p* = 0.015 95% CI: 0.10, 0.70	-		
BBS	r = −0.696 ** p < 0.001 95% CI: −0.84, −0.46	r = −0.264 *p* = 0.159 95% CI: −0.61, 0.12	r = −0.454 *p* = 0.012 95% CI: −0.69, −0.12	r = −0.596 ** p < 0.001 95% CI: −0.82, −0.31	-	
Self-reported global activity level	r = −0.407 *p* = 0.026 95% CI: −0.−66, −0.09	r = −0.215 *p* = 0.254 95% CI: −0.55, 0.17	r = 0.545 * p = 0.002 95% CI: −0.79, −0.24	r = 0.521 * p = 0.003 95% CI: −0.76, −0.18	r = 0.451 *p* = 0.012 95% CI: 0.10, 0.70	-

HADS-A, Hospital Anxiety and Depression Scale—Anxiety; HADS–D, Hospital Anxiety and Depression Scale—Depression; PDQ-39, Parkinson’s Disease Questionnaire; FTSTS, Five-Time Sit-to-Stand Test; BBS, Berg Balance Scale. CI, confidence interval. Note. Correlation coefficients represent Spearman’s r values. Statistical significance was evaluated using Bonferroni correction for six comparisons (adjusted α = 0.0083). * *p* < 0.008, ** *p* < 0.001.

## Data Availability

The data presented in this study are available upon request from the corresponding author due to their being part of an ongoing study.
